# Perceived Riskiness and Problem Gambling Across Different Forms of Gambling: A Focus on 'Soft' Gambling

**DOI:** 10.1007/s10899-024-10370-y

**Published:** 2024-12-28

**Authors:** David Fiedor, Miroslav Charvát, Filip Kovařík, Jindřich Frajer, Eva Aigelová, Miloslav Šerý

**Affiliations:** 1 Department of Geography, Faculty of Science, Palacký University Olomouc, Olomouc, Czech Republic; 2 Department of Psychology, Faculty of Arts, Palacký University Olomouc, Olomouc, Czech Republic

**Keywords:** Experience, Gambling, Lottery, Perceived riskiness, Soft gambling, PGSI short-form

## Abstract

Gambling encompasses a wide variety of activities, and the structural characteristics of each form contribute to its potential risk. However, the literature does not fully agree on the risk levels of certain gambling forms. In this study, we classify less risky gambling forms (soft forms) based on public perceptions of their riskiness. We examine the link between gambling experience and problem gambling prevalence. A survey was conducted in a model region of the Czech Republic, a post-socialist country with high gambling availability, with N = 2,498 respondents. A typology of gambling forms (lotteries, betting, and casino games) was created based on perceived risk similarities. Lotteries are the most frequently played gambling form, with 86.3% reporting lifetime participation. Among those who exclusively engage in lottery-type forms, 15 percentage points more women than men participated in the last year, and the gap widens to 31 points over a lifetime. Forms of gambling perceived as more risky show a lower proportion of non-problem gamblers, both for recent and lifetime participation. Furthermore, individuals who gambled within the past month or year are at higher risk of developing gambling problems compared to those whose gambling experiences were less recent.

## Introduction

Gambling can serve as a leisurely pastime for some, while for others, it poses a severe problem (Latvala et al., 2019). A significant portion of the literature is dedicated to studies focusing on the identification of factors associated with the development of problematic or pathological gambling (Welte et al., 2004). Typically, authors classify these factors into structural and situational characteristics (Parke & Griffiths, 2006). While situational characteristics are deemed crucial in influencing the initial decision to start gambling (Blaszczynski, 2013), structural characteristics affect subsequent gambling behaviour. This behaviour can be influenced by the nature of individual games of chance, which vary in terms of stake amounts and volatility, probability of winning, jackpots, game speed, the presence of so-called near miss events, as well as auditory and visual effects, all of which can potentially sway player behaviour (Gooding & Williams, 2023). Consequently, different forms of games present varying levels of risk for the development of problematic gambling (Mravčík et al., 2020).

Although attention has already been paid to the level of risk of some forms of gambling (Delfabbro et al., 2020; Mazar et al., 2020), there is no sharp dividing line between less risky (soft) and riskier (hard) forms of gambling. There is widespread agreement that certain forms of gambling can be clearly distinguished in terms of their risk levels. Electronic Gambling Machines (EGMs) and live casino games, such as roulette or blackjack, are typically classified as riskier (or 'hard') forms of gambling. In contrast, lotteries are generally considered less risky (or 'soft') forms. This categorisation is widely supported in the academic literature (Booth et al., 2021; Russell et al., 2023). Nonetheless, there is a lack of consensus within the academic community regarding the classification of certain gambling activities, such as scratch cards, sports betting, and bingo, where divergent perspectives prevail. In particular, sports betting has been a point of contention; while some researchers categorise it as a relatively low-risk activity (Binde et al., 2017), others argue that it presents a higher risk (Booth et al., 2021). Moreover, the accelerating digitalization of gambling markets (Ghelfi et al., 2024), enabling participation at virtually any time and location (Gainsbury, 2015), suggests that the risk levels of individual gambling activities may be evolving.

Previous research on the risks associated with different forms of gambling has predominantly focused on the structural characteristics of these games. However, it is crucial to also consider this issue from the perspective of the general population to gain a better understanding of how they perceive the risks and consequences related to gambling. The perception of a game's riskiness in the general population can mirror its actual risk level. Several key factors influence public perception. First and foremost are the regulatory and legislative measures implemented by the governments, which, in general, can be more liberal or restrictive towards gambling (Gavriel-Fried et al., 2023), potentially limiting the availability of specific forms of gambling (Engebø et al., 2021), and thus creating a framework for the social normalisation of certain gambling activities. This normalisation can also be reinforced by sponsorship or permitted advertising from gambling companies (Salonen et al., 2018; Torrance et al., 2021). State policy towards gambling can lead people to carefully weigh the negatives and positives of participating in these games (Lerkkanen et al., 2020), while at the same time, public opinion can help activate state policies aimed at reducing the most risky gambling activities (Booth et al., 2021). The perception of the riskiness of specific games can also be influenced by problems that gambling has caused to family members, acquaintances, and friends, such as an increased likelihood of developing problem gambling (Dowling et al., 2017) or the threat of social exclusion (Takiguchi et al., 2022). It is, of course, necessary to consider individual personality factors (Chalmers & Willoughby, 2006), attitudes towards risk-taking and liberalism (Chiu & Storm, 2010), as well as the developmental perspective of the individual, as the perception of different gambling activities may change with age (Reith & Dobbie, 2012).

Differing perceptions of riskiness among gamblers and the general population can be both a protective and a risk factor, determining the level of involvement in a particular form of gambling. Risk perception of various forms of gambling has been the primary concern of recent studies in Australia, which have focused on the perceived risks associated with four specific games of chance among women (McCarthy et al., 2018), adolescents and adults (Thomas et al., 2017), and in an expanded sample of nine games of chance in the adult population (Booth et al., 2021). Perceptions of the riskiness of specific games among problem gamblers have recently been investigated in Canada by Gooding and Williams (2023). However, research addressing these dynamics in Europe, particularly post-socialist regions, is lacking. Given the Czech Republic's unique history, especially the significant growth in gambling after the fall of communism in 1989, our study focuses on this region to fill the existing gap in European research and to better understand the local gambling landscape. Despite recent legislative reforms, gambling in the Czech Republic remains highly accessible compared to other EU countries (Fiedor et al., 2017). This increased availability, however, has led to socially negative consequences, which in turn has reinforced the Czech population's generally negative attitudes towards gambling. These attitudes are notably more negative in comparison to other countries. Interestingly, certain forms of gambling, such as lotteries, scratch cards, or bingo, are not even considered gambling activities by the majority of the general population (Fiedor et al., 2019a). In the Czech Republic, more than half of the population (56.5%) has a lifetime experience with gambling, most commonly with lotteries and scratch cards (49.1%). In the past 12 months, 28.1% of adults participated in gambling, with 22% engaging in lotteries and scratch cards and 6.3% in sports betting. These participation rates suggest that some forms of gambling may be perceived as less risky and more socially acceptable. However, how might these activities influence the development of problem gambling? According to the Problem Gambling Severity Index (PGSI), 5.8% of the population was at risk, with 1.9% in the problem gambler category (Chomynová et al., 2024). This paper aims to examine the level of problem gambling among players of gambling forms that are generally perceived as less risky in the Czech population in relation to other forms. To achieve this goal, it is also necessary to create a typology of forms of gambling based on their perceived riskiness by the adult population.

## Methods

### Research Design

We employed a questionnaire targeting the adult population^1^ in the Czech Republic. Data collection took place in the Olomouc region, chosen as a model region due to its diversity, encompassing urban agglomerations, rural areas, and peripheral regions (Fiedor et al., 2019a). The region is also suitable for this study concerning gambling availability, as it includes a casino resort that hosts large poker tournaments, as well as other forms of gambling like betting shops and lotteries. In contrast, gambling is scarcely present in peripheral areas (Frajer et al., 2024).

The questionnaire distribution was conducted both on paper (PAPI) in the field (June 2023) and online (second half of 2023). For the field survey, pre-trained university students acted as interviewers, visiting various municipalities in the Olomouc region. Subsequently, a list of mayors in the Olomouc region was compiled, and a two-round system was used to approach them with a request to share the questionnaire on their municipalities' websites or social media. Out of 402 municipalities, 123 responded positively, representing a 31% response rate. Given the high workload of mayors (Bakoš et al., 2021; Fiedor et al., 2019b), we consider this return rate to be high. The questionnaire was also shared by official institutions (Olomouc Region) and some regional media (newspapers, radio). As a result, respondents came from 277 municipalities, representing nearly 69% of the total.

### Questionnaire Components

The questionnaire included socio-demographic questions: gender (male, female, and other), age, highest educational attainment, and respondent's municipality of residence. The further content focused on the perception of the risk associated with specific forms of gambling and personal experience with them. The specific forms of gambling included the detailed breakdown into 12 categories: land-based and online numerical lotteries, paper and online scratch cards, land-based and online sports betting, live sports betting, land-based and online slot machines, live casino games, and land-based and online poker and card tournaments. The perceived risk of each form was measured using a three-point ordinal scale: 1 = low risk, 2 = medium risk, and 3 = high risk. Personal experience or exposure to the presented forms of gambling was measured using standardised questions based on face validity, with clear wording recommended as best practice (Williams & Volberg, 2012) and commonly employed in population-wide studies (Chomynová et al., 2023; Mokinaro et al., 2020). For each of the 12 categories, the participants were asked whether they had engaged in the activity within the last month (LMP—Last Month Prevalence), the last year (LYP—Last Year Prevalence), at any time during their lifetime (LTP—Lifetime Prevalence) or never.

The questionnaire concluded with a brief screening instrument for identifying problematic gambling, selected for its diagnostic accuracy in relation to LYP (Dowling et al., 2019). Specifically, the PGSI Short-Form (Volberg & Williams, 2012) was employed, which consists of three statements regarding financial difficulties caused by gambling, the impact of gambling on relationships, and feelings of guilt related to gambling behaviour. Responses were recorded on a four-point ordinal scale: 0 = never, 1 = sometimes, 2 = most of the time, and 3 = almost always. The sum of the scores produces a total raw score, which categorises respondents into four groups: non-problem (0 points), low risk (1 point), moderate risk (2–3 points), and problem gambler (4 or more points). Conducting confirmatory factor analysis (CFA) on our sample for a three-item scale is not feasible in a meaningful way due to the limited number of items. However, internal consistency, measured by Cronbach's alpha, reached a value of 0.80, indicating acceptable reliability. The average inter-item correlation was 0.57. The PGSI Short-Form, in its Czech version, was also used by Chomynová et al., (2023, 2024) at the National Monitoring Centre for Drugs and Addiction to monitor gambling behaviour in the Czech Republic.

### Participants

A total of 2,724 completed questionnaires were collected. After the cleaning process, 226 questionnaires were excluded (5.5% from the paper form and 11.6% from the online form) due to invalid completion (127), respondent being underage (55), or the respondent not belonging to the area under study, the Olomouc region (44). This resulted in a final number of 2,498 analysed questionnaires, of which 1,388 (55.6%) were paper-based and 1,110 (44.4%) were online. The final sample included 1,047 (41.9%) men, 1,436 (57.5%) women, and 15 (0.6%) respondents who identified as another gender or did not specify their gender. The mean age was 43.1 years (Md = 41, Min = 18, Max = 93). The sample represents 0.5% of the adult population in the region. A detailed description of the participant sample and comparison with the target population (CZSO, 2023) is provided in Table 1.

**Table 1:** Comparison of basic socio-demographic data of the population and the sample

	N (%)	Gender			Education			
	18 years and older	Men	Women	Other	ES	HS	CU	N.A
Population of the Olomouc Region (2023)	513,518	248,474 (48.4)	265,044 (51.6)	N.A. (0.0)	64,397 (12.5)	342,084 (66.6)	81,795 (15.9)	25,242 (4.9)
PAPI collection	1,388	581 (41.9)	800 (57.6)	7 (0.5)	119 (8.6)	1,016 (73.2)	235 (16.9)	18 (1.3)
ONLINE collection	1,110	466 (42.0)	636 (57.3)	8 (0.7)	30 (2.7)	630 (56.7)	445 (40.1)	5 (0.5)
Total sample	2,498	1,047 (41.9)	1,436 (57.5)	15 (0.6)	149 (6.0)	1,646 (65.9)	680 (27.2)	23 (0.9)

ES = Elementary School (No High School Diploma), HS = High School Diploma or Equivalent, CU = College/University Diploma, N.A. = data not available or missing

The study received approval from the Faculty of Arts Ethics Panel at Palacký University Olomouc (standard FF-B-20–02, registration number 02/2023). Data collection was anonymous, and participation was entirely voluntary. The participants provided their informed consent to participate in this study. In the field, consent was given orally, and in the online form, participation was confirmed by clicking the ‘fill out the questionnaire’ button. The research was conducted in compliance with the ethical code of the European Federation of Psychologists' Associations (EFPA, 2005).

### Analysis

The analyses used to process the questionnaire data are divided into two parts, reflecting the structure of the results section: (1) perception of the risk associated with forms of gambling, and (2) respondents' experiences and the risk of their gambling behaviour.

The first part of the results aimed to differentiate forms of gambling in terms of their perceived risk. To accomplish this goal, we performed a cluster analysis of items assessing the perceived riskiness of different forms of gambling, rather than clustering the participants themselves. For the analysis, polychoric correlations were used, addressing the problem of ordinal items and allowing the application of standard procedures using correlations between variables, such as clustering or factor analysis. The polychoric correlation matrix thus formed the input data for cluster analysis using the ‘iclust’ function from the ‘psych’ package in the R program (Revelle, 2024). Given the ambiguity of the results, especially in terms of the number of resulting clusters, the EGA—Exploratory Graph Analysis (EGA) method was also used via the EGAnet package in R (Golino & Christensen, 2024), again with the polychoric correlation matrix as input. While both methods serve to identify patterns in the data, EGA offers a complementary perspective by applying community detection algorithms to analyse variable interconnections. Unlike traditional clustering, which focuses primarily on grouping entities, EGA's strength lies in its ability to uncover underlying factor structures, potentially providing additional insights into the ambiguous clustering results.

The second part of the results focused on grouping respondents into mutually exclusive groups based on their gambling experience. Respondents were examined to determine if they had played at least one game of a particular form (defined by the riskiness of the gambling form—see the previous section) in the last month, in the last year, or at least once in their lifetime. For classification purposes**,** if a respondent had played at least one specific form of gambling from a given cluster with a predetermined frequency, they were classified as a player of that gambling form. Given the number of combinations of game categories, the rule applied was that a gambling form with higher perceived riskiness is a dominant indicator of problematic gambling behaviour.

## Results

### Perception of Risk in Gambling Forms

**Fig. 1:**
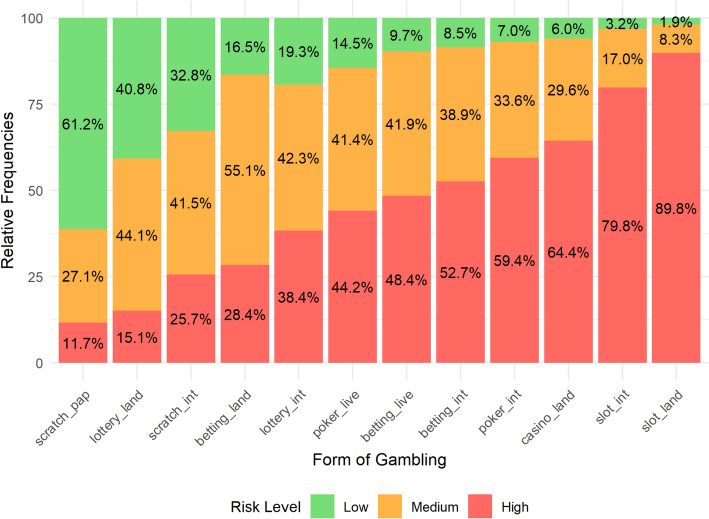
Perceived Risk Level Distribution for Various Forms of Gambling. lottery_land = Land-based Lottery/Retail Lottery, lottery_int = Internet Lottery/Online Lottery, scratch_pap = Paper Scratchcards/Physical Scratchcards, scratch_int = Internet Scratchcards/Online Scratchcards, betting_land = Land-based Betting/Retail Betting, betting_int = Internet Betting/Online Betting, betting_live = Live Betting/In-play Betting, slot_land = Land-based Slot Machines/Physical Slot Machines, slot_int = Internet Slot Machines/Online Slots, Casino_land = Land-based Casino Games/Casino Table Games, poker_live = Live Poker/In-person Poker, poker_int = Internet Poker/Online Poker

As outlined in previous chapters, different forms of gambling vary significantly from one another. Therefore, it was anticipated that their perceived risk levels would also differ. This assumption is confirmed by Fig. 1. The only form of gambling that the majority of respondents (61.2%) perceive as having a low risk of developing problem gambling is paper scratch cards. On the other hand, online betting, online poker, casino table games, and Electronic Gambling Machines (EGMs), whether in land-based venues or online, are viewed by more than half of the respondents as high-risk forms of gambling.

**Fig. 2:**
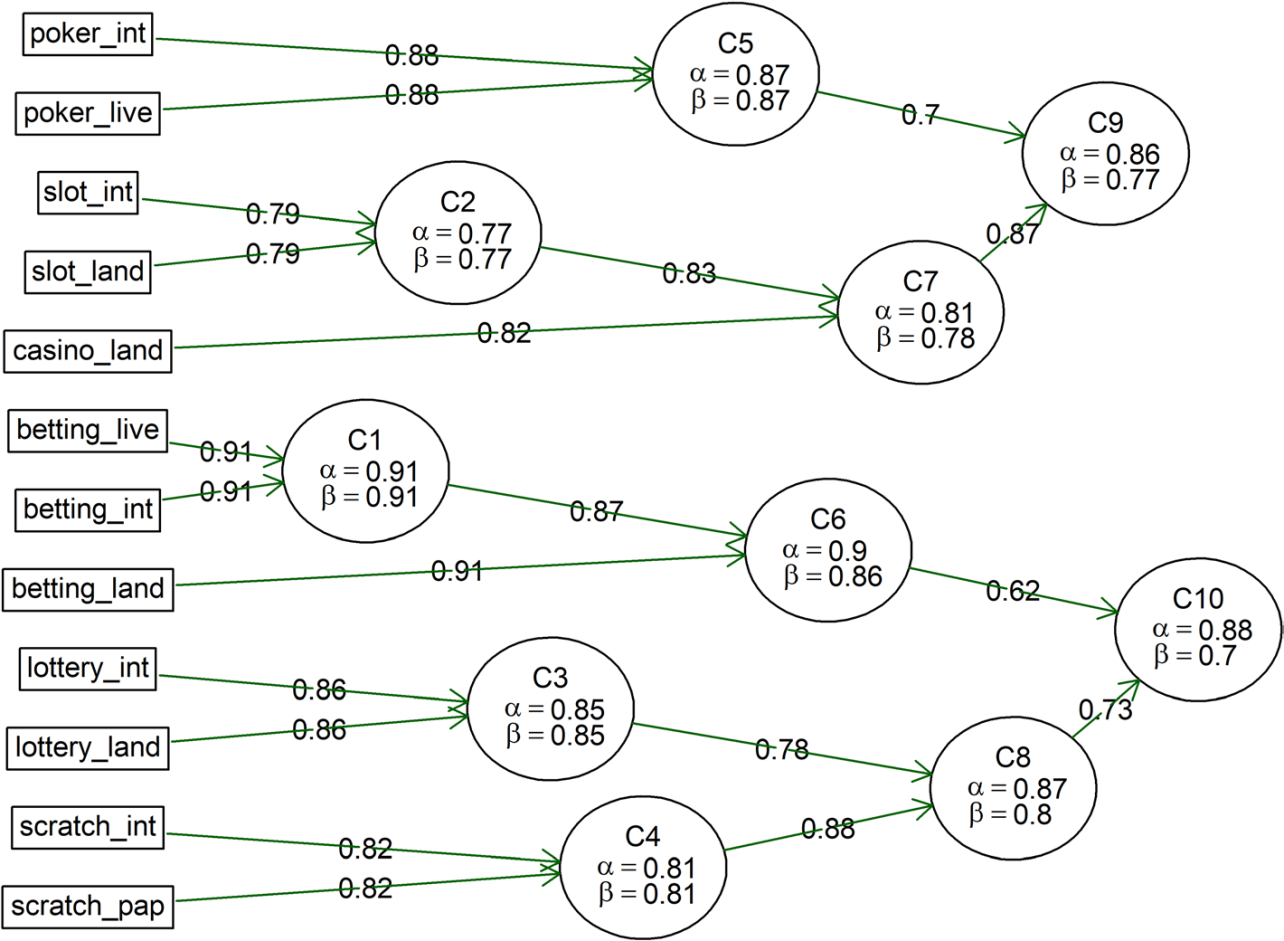
Forms of Gambling Clustered by Perceived Level of Risk (Two-Cluster Solution). lottery_land = Land-based Lottery/Retail Lottery, lottery_int = Internet Lottery/Online Lottery, scratch_pap = Paper Scratchcards/Physical Scratchcards, scratch_int = Internet Scratchcards/Online Scratchcards, betting_land = Land-based Betting/Retail Betting, betting_int = Internet Betting/Online Betting, betting_live = Live Betting/In-play Betting, slot_land = Land-based Slot Machines/Physical Slot Machines, slot_int = Internet Slot Machines/Online Slots, Casino_land = Land-based Casino Games/Casino Table Games, poker_live = Live Poker/In-person Poker, poker_int = Internet Poker/Online Poker

**Fig. 3:**
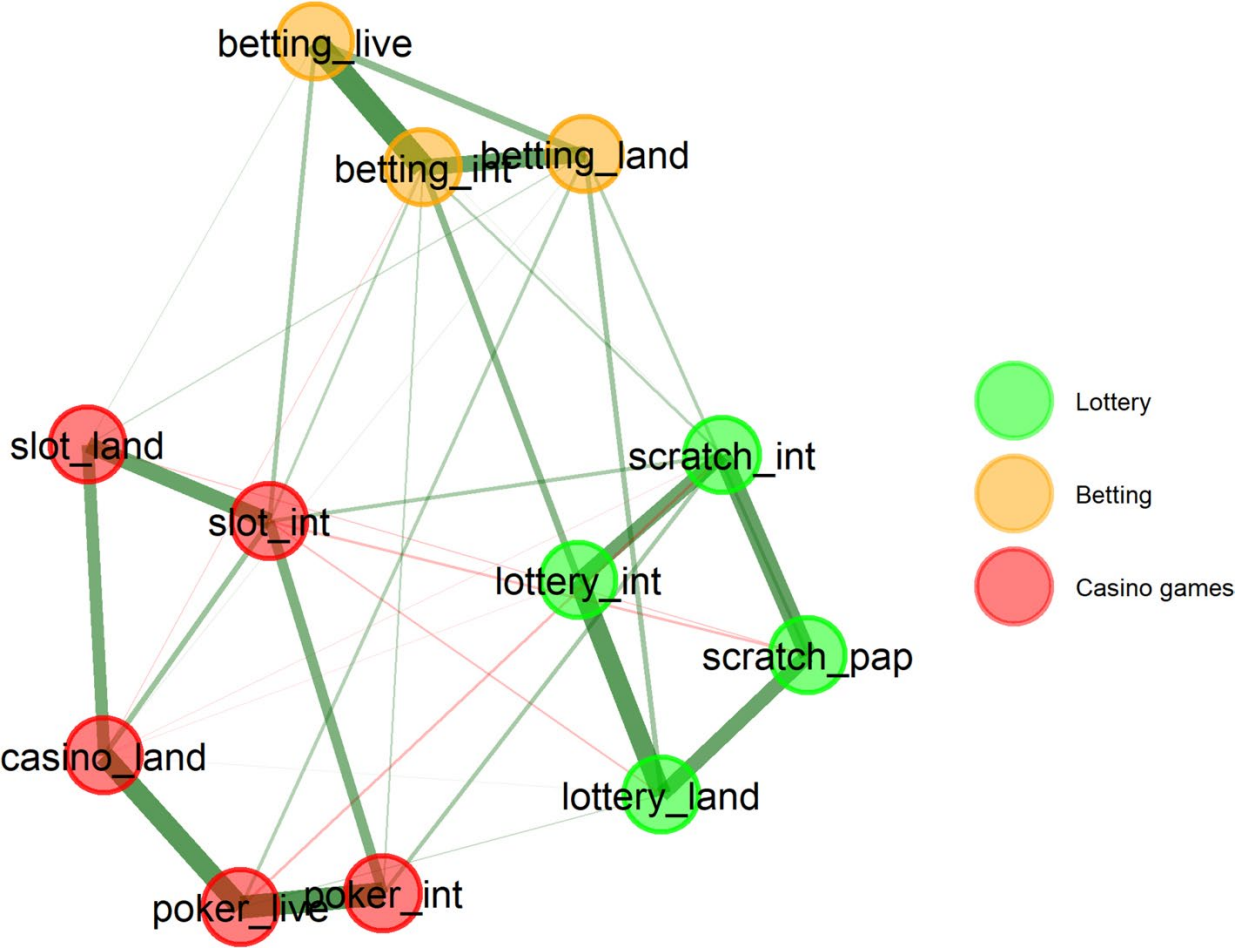
Exploratory Graph Analysis Network Plot of Relationships between Game Types. lottery_land = Land-based Lottery/Retail Lottery, lottery_int = Internet Lottery/Online Lottery, scratch_pap = Paper Scratchcards/Physical Scratchcards, scratch_int = Internet Scratchcards/Online Scratchcards, betting_land = Land-based Betting/Retail Betting, betting_int = Internet Betting/Online Betting, betting_live = Live Betting/In-play Betting, slot_land = Land-based Slot Machines/Physical Slot Machines, slot_int = Internet Slot Machines/Online Slots, Casino_land = Land-based Casino Games/Casino Table Games, poker_live = Live Poker/In-person Poker, poker_int = Internet Poker/Online Poker

Figure 1 also shows that the proportion of respondents identifying a high level of risk increases almost ‘linearly’, while the proportion of low-risk games decreases ‘exponentially’.^2^ Consequently, it is impossible to create distinctly separate categories or types based solely on the distribution of perceived risk frequencies for individual forms of gambling. Given the similarities in perceived risk, a typology of gambling forms was developed (see Fig. 2)—notably, land-based and online variants of individual forms are always clustered together first. Although there are some differences in the perception of risk between online and land-based forms of gambling, their risk assessments are closely related. When examining the factor loadings of the two resulting clusters (Table 2), it is evident that Cluster C10, formed from Clusters C6 and C8, is more heterogeneous than Cluster C9. Therefore, it is worth considering whether to account for three clusters instead of two. This notion is further supported by another clustering model using Exploratory Graph Analysis presented in Fig. 3.

**Table 2:** Factor loadings of various forms of gambling for two and three-cluster solutions

Form of gambling	Cluster	C10 loadings	C9 loadings	Cluster	C9 loadings	C8 loadings	C6 loadings
lottery_land	C10	**0.74**	0.18	C8	0.18	**0.83**	0.45
lottery_int	C10	**0.81**	0.21	C8	0.21	**0.85**	0.56
scratch_pap	C10	**0.69**	0.06	C8	0.06	**0.81**	0.38
scratch_int	C10	**0.76**	0.20	C8	0.20	**0.81**	0.50
betting_land	C10	**0.75**	0.40	C6	0.40	0.51	**0.86**
betting_int	C10	**0.81**	0.37	C6	0.37	0.55	**0.94**
betting_live	C10	**0.71**	0.41	C6	0.41	0.46	**0.85**
slot_land	C9	0.22	**0.65**	C9	**0.65**	0.08	0.34
slot_int	C9	0.32	**0.74**	C9	**0.74**	0.17	0.43
casino_land	C9	0.14	**0.81**	C9	**0.81**	0.06	0.21
poker_live	C9	0.26	**0.82**	C9	**0.82**	0.17	0.32
poker_int	C9	0.37	**0.80**	C9	**0.80**	0.26	0.41

All cluster loadings with a value of 0.6 and above are given in bold

lottery_land = Land-based Lottery/Retail Lottery, lottery_int = Internet Lottery/Online Lottery, scratch_pap = Paper Scratchcards/Physical Scratchcards, scratch_int = Internet Scratchcards/Online Scratchcards, betting_land = Land-based Betting/Retail Betting, betting_int = Internet Betting/Online Betting, betting_live = Live Betting/In-play Betting, slot_land = Land-based Slot Machines/Physical Slot Machines, slot_int = Internet Slot Machines/Online Slots, Casino_land = Land-based Casino Games/Casino Table Games, poker_live = Live Poker/In-person Poker, poker_int = Internet Poker/Online Poker

In light of the above, the authors decided to work with three clusters of gambling forms. The first cluster includes lotteries and scratch cards (both land-based and online), which are considered the least risky from the perspective of perceived risk. Given that scratch cards are a type of instant lottery, this cluster will be referred to as ‘lottery’. The next cluster comprises ‘betting’ (land-based, online, and live), which can be classified between the first and third types in terms of risk. The final, most risky (hard) cluster includes casino table games, EGMs, and poker (again, both land-based and online). Since all these games can be played in a casino, this cluster will be referred to as ‘casino games’. Next, we will focus on the first 2 clusters ('lottery' and 'betting'), which are perceived as less risky forms of gambling.

### Experience with Soft Forms of Gambling and Problem Gambling

**Fig. 4:**
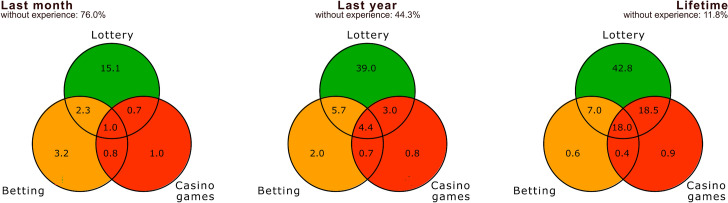
The percentage distribution of respondents' experiences with different types of games (lottery, betting, and casino games) in the last month, last year, and over their lifetime

When assessing respondents' experiences with gambling, the temporal component is a crucial criterion. Lifetime experience with some form of gambling is reported by 88.2% of respondents; 55.7% had gambling experience within the last year, and only 24.0% in the last month (see Fig. 4). The most frequently played form of gambling is unequivocally the lottery, regardless of the time frame. Lifetime experience with lotteries is reported by 86.3% of all respondents. At a significance level of α = 0.05, the null hypothesis of independence between the type of gambler (determined based on their experiences in the last month, last year, and lifetime) and gender^3^ or highest educational attainment was tested using the χ2 test.^4^ In all cases, the null hypothesis of variable independence was rejected, although the sample size played a significant role. The following commentary highlights the most significant deviations along with the adjusted residuals (AR). When focusing on respondents who have only experienced lottery-type games (and no other, more risky forms of gambling), it can be noted that for last year's experiences, there are 15 percentage points more women than men (AR 7.9), and for lifetime experiences, this difference is even 31 percentage points (AR 15.6). The differences in highest educational attainment among lottery players are relatively small and statistically insignificant, except for monthly experience—where high school graduates are more represented (AR 2.9) and university graduates less so (AR −3.0). Compared to other types, lottery players tend to be older (it can be stated that the average age of respondents with experience in more risky forms of gambling—betting, casino games—decreases gradually).

Respondents who belong to the ‘betting’ category (either having experience with betting or also with lotteries) form a very specific category that remains relatively stable over time. This category is dominated by men, particularly when focusing on experiences within the last month (11.5% of men, while only 1.3% of women fall into this category, a difference of approximately 10 percentage points; AR 11.0). The same trend is observed when looking at yearly experiences (AR 10.1) and lifetime experiences (AR 5.6), where the proportion of men is statistically significantly higher. Only a tiny percentage of women play purely betting games and nothing else: 0.5% in the last month, 0.5% in the last year, and only 0.2% in the lifetime. Similarly to lotteries, when evaluating the highest educational attainment, high school graduates are statistically significantly more likely to play betting games (AR 2.7) and university graduates less so (AR −2.6).

**Fig. 5:**
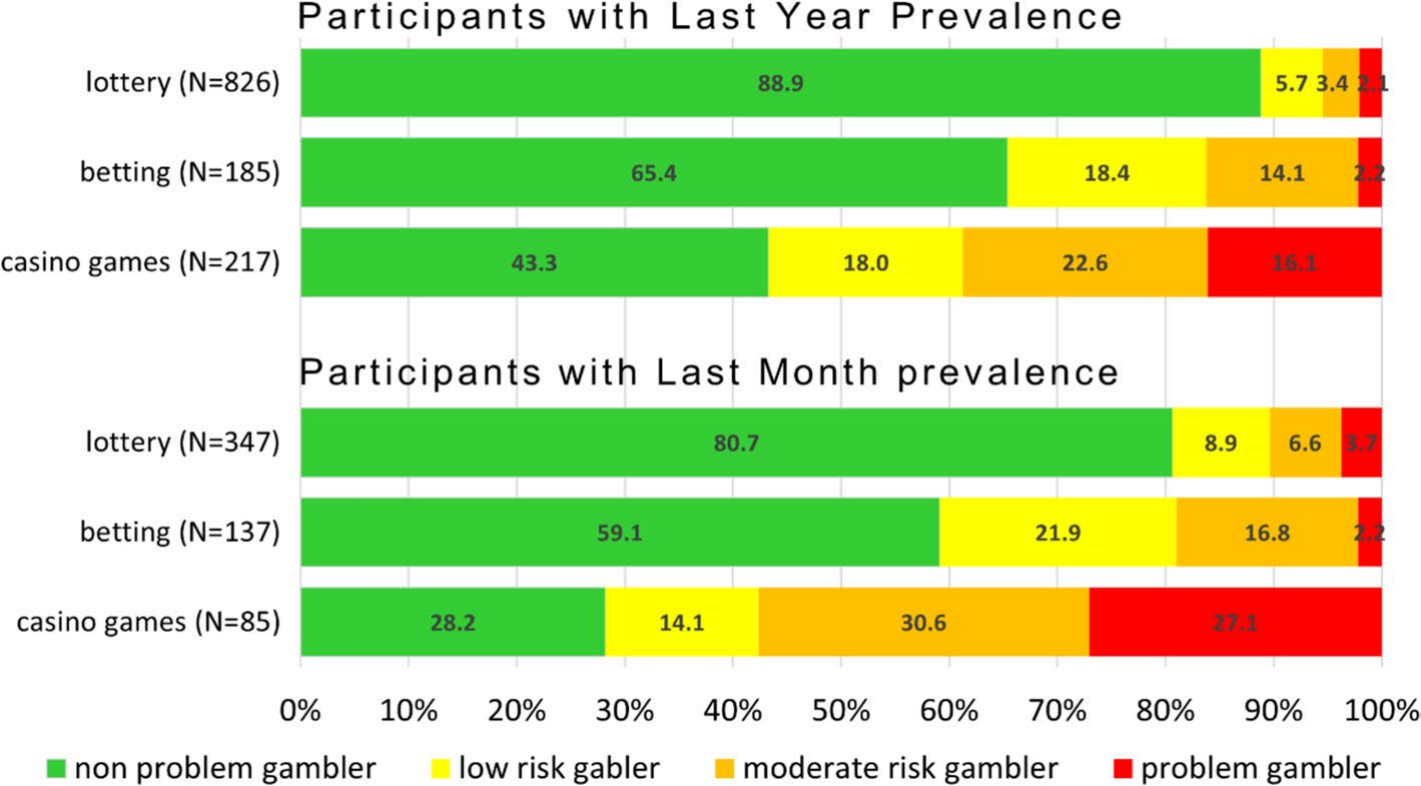
The percentage distribution of problem gambling in categories of respondents who have experience with lotteries, betting, or casino games, both in the last month and year

Using the PGSI-Short Form tool, 67 respondents were classified as problem gamblers, representing a prevalence rate of 2.7%. Problem gambling is clearly linked to players' experiences. When divided into specific types, it is evident that the riskier the form of gambling, the smaller the proportion of non-problem gamblers, both in the last month and the last year (see Fig. 5). Furthermore, when comparing the evaluation of ‘last month’ and ‘last year’ experiences, it is clear that when focusing on players with more recent or regular experiences, the proportion of those at risk of developing problem gambling is higher than when evaluating experiences over the last year.

## Discussion

### Perception of Risk Across Gambling Forms

Cluster analysis of risk perceptions associated with different forms of gambling reveals that the general adult population in the Czech Republic perceives significant differences among these forms. The position of scratch cards and lotteries as the least risky, as perceived by respondents, aligns with findings from studies conducted on adult populations in Australia (Booth et al., 2021), where these activities were considered risky by only 30% and 32% of respondents, respectively. Similarly, bingo, which 24% of Australian respondents considered less risky, is not currently legally operated in the Czech Republic and, therefore, was not included in our study. Casino games, particularly Electronic Gambling Machines (EGMs), are perceived by the public as the most risky, confirming trends highlighted not only by Booth et al. (2021) but also by other studies, such as those by McCarthy et al. (2018) and Thomas et al. (2017). These studies also support the boundary positioning of sports betting, which is perceived as more risky than lotteries and scratch cards but less risky than casino games and EGMs. Within our study, different forms of sports betting also formed a distinct cluster. This confirms the position of sports betting among forms of gambling, which is not explicit, with some authors assigning it to less risky forms (Griffiths, 2002), while others assign it to more risky forms (Noble et al., 2022) and others place it somewhere in between (Meyer et al., 2011).

In contrast to the studies mentioned above, our research separately included online and land-based forms of gambling. Apart from casino games, respondents perceived online versions of identical games as slightly more risky. Public opinion thus broadly aligns with empirical studies indicating that online gambling is becoming the most harmful category (Hing et al., 2022; Marionneau et al., 2024). From a risk perspective, online games of chance (including lotteries) pose a higher risk for the development of problem gambling than their land-based counterparts. Online games are available 24/7, allow for simultaneous play of multiple games, offer instant gameplay, and involve betting or draws that occur with high frequency (Chomynová et al., 2023).

### Soft Forms of Gambling and Their Normalisation

Soft forms of gambling—represented in our research by a cluster of online and offline lotteries and scratch cards—are among the most widespread and accessible forms of gambling activities (Griffiths, 2000). Our findings support this, as these forms of gambling showed the highest participation rates among respondents over monthly (20%), yearly (52%), and lifetime gambling (86%). Our methodology allowed us to filter out players who had engaged in other forms of gambling over the past year, potentially skewing statistics related to problem gamblers who combine different gambling forms (Scalese et al., 2016). Among respondents who only engaged in soft forms of gambling (lotteries and scratch cards) in the last year or month, the percentage of those identified as low, moderate, or problem gamblers according to the PGSI Short-Form was indeed the smallest. However, given the widespread participation in these activities, the absolute number of problem gamblers and gamblers at some level of risk was the highest in absolute terms, regardless of monthly or yearly experience. This result is alarming and could lead to underestimating these games of chance, which, as shown in the study by Maurício and Rodrigues-Silva (2023), can become very dangerous. Despite their risks, these forms of gambling are often overlooked, as empirical studies suggest that EGMs, casino games, and sports betting are more strongly associated with problem gambling than lotteries or scratch cards (Binde et al., 2017). Our study also identified scratch cards as the least risky (see Fig. 1). Their status as harmless entertainment is often normalised within families, where scratch cards are frequently given as Christmas or birthday gifts (even to juveniles), according to SAZKA a.s. (largest scratch cards and lottery operator in the Czech Republic) 7 out of 10 adults engage in scratching cards during Christmas and gain the biggest daily revenue in this field (Czech Television, 2024). Research by Kundu et al. (2013) confirmed that receiving a scratch card as a gift during childhood is more often associated with problem gambling and a positive attitude towards gambling in general. In the context of gambling development in the Czech Republic, the normalisation of soft forms (particularly among the older generation) is reinforced by the fact that state-run lotteries were the only permitted and accessible form of gambling during the socialist era, while other forms were banned (Fiedor et al., 2019a). Currently, the offer of gambling in the Czech Republic remains high. However, while the new Gambling Act (2017) and local regulations have led to a reduction in land-based venues offering EGMs and casino games (Frajer et al., 2024), the sale of lotteries (both traditional and instant scratch cards) is not legislatively restricted beyond customer age (18 years). Lottery tickets and scratch cards can be purchased in ordinary grocery stores, newsstands, and post offices. Rodrigues-Silva (2017) previously highlighted a similar situation of unregulated and widely available gambling in Portugal, calling it a ‘hidden threat’. Additionally, sellers often do not adhere to the rule of minimum age (Griffiths, 2000; Rodrigues-Silva, 2017), leading to distribution among adolescents and juveniles. This is confirmed by the international ESPAD study, where scratch cards and lotteries were the most common gambling experiences among the Czech school population (Chomynová et al., 2020).

### Gender Differences and Problem Gambling Prevention

Our research on the adult population revealed that women are statistically significantly more represented among players of soft forms of gambling, with greater experience on both a yearly and monthly basis. It thus becomes evident that the involvement of women in various forms of gambling activities is not solely the domain of men, and the number of women participating in gambling activities is increasing (Thomas et al., 2017). However, this increase may also lead to a rise in the number of problem female gamblers, whose gambling behaviour and patterns differ from those of men. Certain soft forms of gambling, such as bingo or scratch cards, are particularly popular among women (Wenzel & Dahl, 2009). Nower and Blaszczynski (2006) report that among problem female gamblers, scratch-off lottery tickets, along with EGMs, were among the introductory gambling activities. Furthermore, Gooding and Williams (2023) found that, in their research among problem gamblers, besides the dominant EGMs, lotteries or raffle tickets, along with instant lotteries, were most frequently identified as gambling activities that caused them the most problems. Regarding problem gambling, there is no clear-cut dichotomy between ‘problem gamblers’ and ‘non-problem gamblers’. Research suggests that the issue is better understood as a continuum of problems and describes the so-called prevention paradox in terms of the distribution of prevalence of harm from gambling in the population (Browne & Rockloff, 2017, 2018; Delfabbro & King, 2019; Delfabbro et al., 2020). This public health concept explains the distribution of symptoms or consequences of a condition within a population. Quantitatively, the majority of the negative consequences of gambling fall on the less severe cases rather than the more severe ones, simply because there are far more of the less severe cases. From an individual perspective, the priority is the intervention or treatment of more severe cases with more intense manifestations of problem gambling. When assessing the severity of problem gambling using the PGSI Short Form, it functions solely as a screening tool and lacks the diagnostic precision of an evaluation by a psychiatrist or addiction specialist. However, many individuals with gambling problems, particularly in the Czech Republic, delay seeking treatment. There is typically a 2–5 year gap between the onset of serious issues and entering a treatment program (Maierová et al., 2014), and the number of treated cases falls significantly short of the estimated prevalence of problem gambling in the population (Chomynová et al., 2024). Therefore, relying on validated screening is more suitable for our study than focusing solely on individuals with an official diagnosis. However, the concept of the preventive paradox refers to the greater societal benefit achieved by slightly reducing the harm from risky gambling patterns across a much larger group of at-risk gamblers with less severe problems than by attempting to minimise issues among a smaller number of problem (high-risk) gamblers. This is especially true given that interventions for players with already developed problems are much more challenging, even financially. From a prevention standpoint and within a public health approach, it is recommended to focus on the entire gambling population (Hancock & Smith, 2017; Livingstone & Rintoul, 2020), as gambling is always a risky activity. For this reason, increased attention should also be paid to soft forms of gambling and their spatial and social availability.

### Potential Methodological Limits of the Study

The study may have several potential limitations. In all cases, the authors endeavoured to avoid the influence of specific methodological choices on the study results. Primarily, this concerns the combination of two different questionnaire data collection methods: PAPI and online. The aim was to capture the full breadth of the population and reflect both known and unknown characteristics within it. Although the perception of risk for individual gambling forms differed between the two subpopulations, the same types of gambling (lottery, betting, casino games) emerged in both samples based on perceived risk. When dividing the respondents into those approached via PAPI, we obtained the same clusters of gambling forms as with respondents who completed the questionnaire online. There were no 'method effects' across the two different recruitment strategies.

In cluster analyses, the selection of specific methods, parameter settings, and determination of the final number of clusters are always crucial considerations. Within clustering methods, several approaches suitable for ordinal data are available, as the perceived riskiness of gambling forms was examined on a three-point ordinal scale (the choice of three levels—low, medium, and high risk—may be limiting in itself; however, we preferred this setting for its comprehensibility and simplicity). In the paper, we presented two specific methods with default settings: cluster analysis using the 'iclust' function and the EGA function from the 'EGAnet' package (described in detail in the Methods section). Additionally, other statistical processing methods were considered, specifically Exploratory Factor Analysis and Non-metric Multidimensional Scaling. In these cases as well, the results were similar, with the three clusters described in the results section providing the most meaningful interpretation.

## Conclusions

Could gambling forms that are generally perceived as less risky still pose a problem for society? It is evident that the proportion of problem gamblers is generally lower for these games of chance. However, it is crucial to consider their widespread societal acceptance and the resulting high level of public experience with these activities. Few people, when imagining someone with a gambling disorder, would not picture a person engaging in EGMs or casino games. However, as it turns out, many lottery players who do not engage in more risky forms of gambling still fall into the category of problem gamblers. It is somewhat regrettable that, both in academic literature and in public discourse, lotteries and other less risky forms of gambling do not receive the attention they warrant. Although these games are a source of entertainment for many, the absolute number of problem gamblers remains significant. This issue may be exacerbated by legislative and preventive measures that fail to adequately highlight the risks associated with these activities, thereby failing to protect players effectively. Monitoring the subjective perception of the riskiness of different forms of gambling is essential for targeted prevention, public education, training of addiction professionals, and personalised treatment. Furthermore, it can serve as a foundation for effective legal regulation of gambling, development of state legislation, and responsible marketing by operators.

## Data Availability

The data that support the findings of this study are available from the corresponding author, [F.K.], upon reasonable request.

## Footnotes

1 In the Czech Republic, participation in gambling is legally permitted only from the age of 18. Therefore, the survey focused solely on the adult population (18 +).

2 It is important to view this in context, as the situation does not represent a single variable and its behaviour, but rather outlines a scenario where there are no ‘jumps’ that would facilitate the straightforward creation of a typology. Instead, the various types of games are arranged according to the proportion of respondents who indicated a high level of risk.

3 Given the very low number of respondents identifying with a gender other than male or female, the analyses were conducted only for men and women.

4 The only exception is a statistically insignificant difference (p-value = 0.054) in the type of players determined by lifetime experience with gambling and highest educational attainment.
